# Redetermination of 1-cyclo­hexyl-3-(2-furo­yl)thio­urea

**DOI:** 10.1107/S1600536810013693

**Published:** 2010-04-17

**Authors:** J. Duque, O. Estévez, V. Jancik, H. Yee-Madeira

**Affiliations:** a Instituto de Ciencia y Tecnología de Materiales, Universidad de La Habana, Cuba; b Instituto de Química, UNAM, Mexico; c Escuela Superior de Física y Matemática, IPN, Mexico

## Abstract

The title compound, C_12_H_16_N_2_O_2_S, was synthesized from furoyl isothio­cyanate and cyclo­hexyl­amine in dry acetone, and the crystal structure redetermined. The thio­urea group is in the thio­amide form. The structure [Otazo-Sánchez *et al.* (2001 ▶). *J. Chem. Soc. Perkin Trans. 2*, pp. 2211–2218] has been redetermined in order to establish the intra- and inter­molecular inter­actions. The *trans*–*cis* geometry of the thio­urea group is stabilized by intra­molecular hydrogen bonding between the carbonyl and *cis*-thio­amide groups, resulting in a pseudo-*S*(6) planar ring which makes a dihedral angle of 3.24 (6)° with the 2-furoyl group and a torsion angle of −84.3 (2)° with the cyclo­hexyl group. There is also an intra­molecular hydrogen bond between the furan O atom and the other thio­amide H atom. In the crystal structure, mol­ecules are linked by inter­molecular N—H⋯O hydrogen bonds, forming chains along [010].

## Related literature

For general background to the applications of aroylthio­ureas in coordination chemistry and mol­ecular electronics, see: Aly *et al.* (2007 ▶); Koch (2001 ▶); Duque *et al.* (2009 ▶); Estévez-Hernández *et al.* (2006 ▶). For related structures, see: Estévez-Hernández *et al.* (2008 ▶). For the synthesis, see: Otazo-Sánchez *et al.* (2001 ▶).
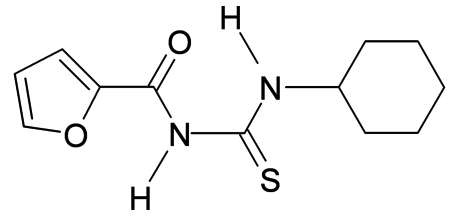


## Experimental

### 

#### Crystal data

C_12_H_16_N_2_O_2_S
*M* *_r_* = 252.33
Orthorhombic, 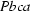

*a* = 7.2667 (5) Å
*b* = 10.2058 (7) Å
*c* = 34.239 (3) Å
*V* = 2539.3 (3) Å^3^
*Z* = 8
Mo *K*α radiation
μ = 0.25 mm^−1^
*T* = 100 K
0.37 × 0.34 × 0.23 mm

#### Data collection

Bruker APEXII CCD diffractometer
Absorption correction: multi-scan (*SADABS*; Sheldrick, 2008 ▶) *T*_min_ = 0.914, *T*_max_ = 0.946
30412 measured reflections
2232 independent reflections
2175 reflections with *I* > 2σ(*I*)
*R*_int_ = 0.019

#### Refinement

*R*[*F* ^2^ > 2σ(*F* ^2^)] = 0.033
*wR*(*F* ^2^) = 0.072
*S* = 1.20
2232 reflections
160 parameters
H atoms treated by a mixture of independent and constrained refinement
Δρ_max_ = 0.25 e Å^−3^
Δρ_min_ = −0.22 e Å^−3^

### 

Data collection: *APEX2* (Bruker, 2007 ▶); cell refinement: *SAINT* (Bruker, 2007 ▶); data reduction: *SAINT*; program(s) used to solve structure: *SHELXS97* (Sheldrick, 2008 ▶); program(s) used to refine structure: *SHELXL97* (Sheldrick, 2008 ▶); molecular graphics: *ORTEP-3 for Windows* (Farrugia, 1997 ▶) and *Mercury* (Macrae *et al.*, 2008 ▶); software used to prepare material for publication: *WinGX* (Farrugia, 1999 ▶).

## Supplementary Material

Crystal structure: contains datablocks global, I. DOI: 10.1107/S1600536810013693/bq2204sup1.cif

Structure factors: contains datablocks I. DOI: 10.1107/S1600536810013693/bq2204Isup2.hkl

Additional supplementary materials:  crystallographic information; 3D view; checkCIF report

## Figures and Tables

**Table 1:** Hydrogen-bond geometry (Å, °)

*D*—H⋯*A*	*D*—H	H⋯*A*	*D*⋯*A*	*D*—H⋯*A*
N1—H1⋯O1	0.83 (2)	2.329 (19)	2.7342 (17)	110.8 (16)
N1—H1⋯O2^i^	0.83 (2)	2.32 (2)	3.0799 (18)	153.0 (18)
N2—H2⋯O2	0.83 (2)	1.983 (19)	2.6574 (18)	138.0 (18)

Symmetry code: (i) 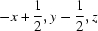.

**Fig. 1.**
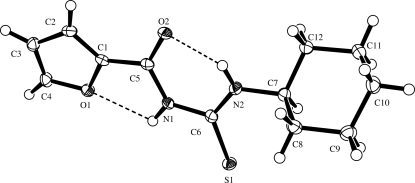
The molecular structure of the title compound. Displacement ellipsoids are drawn at the 50 % probability level. The intramolecular N—H···O hydrogen bonds are shown as dashed lines.

**Fig. 2.**
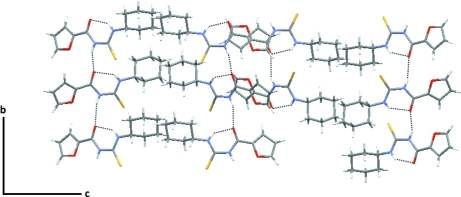
View of the crystal packing of the title compound. Intermolecular hydrogen bonds are shown as dashed lines.

## supplementary crystallographic information

### Comment

Aroylthioureas have applications in metal complexes and molecular electronics (Aly *et al.*, 2007, Duque *et al.*, 2009). Coordination chemistry of such derivatives is more varied than that of simple thiourea and physiochemical properties result in a number of potential technical and analytical applications (Koch, 2001, Estévez-Hernández *et al.*, 2006). The structure of the title compound (I), Fig.1, has been re-determined and the results adds significantly to the information already in the public domain (Otazo-Sánchez *et al.*, 2001), especially about the intra and intermolecular interactions (not reported previously). The data and the refinement of the structure are also of a little better quality (present refinement: R: 0.033 and wR: 0.072; previous refinement: R: 0.031 and wR: 0.082), because it was measured at low temperature (100 °K) to diminish disorder of the atoms in the unit cell. The main bond lengths and angles are are within the ranges obtained for similar compounds (Estévez-Hernández *et al.*, 2006). The C6—S1 and C5—O1 bonds show typical double-bond character. However, the C—N bond lengths, C5—N1, C6—N1, C6—N2 are shorter than the normal C—N single-bond length of about 1.48 Å. These results can be explained by the existence of resonance in this part of the molecule. The central thiourea fragment (N1/C6/S1/N2) makes dihedral angle of 3.24 (6) ° with the 2-furoyl group (O1/O2/C5/C1—C4/) and a torsion angle of -84.3 (2)° with the cyclohexyl group (C6—N2—C7—C8), respectively. The *trans*-*cis *geometry in the thiourea moiety is stabilized by the N2—H2···O2 hydrogen bond (Fig.1 and Table 1). An additional intramolecular hydrogen bond N1—H1···O1 is observed. In the crystal structure symmetry related molecules are linked by N1—H1···O2 interactions to form one-dimensional chains along the *b* axis (Fig. 2 and Table 1).

### Experimental

The title compound, (I), was synthesized according to a procedure described by Otazo-Sánchez *et al.* (2001), by converting furoyl chloride into furoyl isothiocyanate and then condensing with cyclohexylamine. The resulting solid product was crystallized from ethanol yielding X-ray quality single crystals (m.p. 70-71 °C). Elemental analysis for C_12_H_16_N_2_O_2_S found: C 57.28, H 6.18, N 11.08, S 12.36 %; calculated: C 57.14, H 6.35, N 11.11, S 12.70 %.

### Refinement

All H atoms were refined with *U*_iso_(H)=1.2*U*_eq_(C/N).

### Crystal data

**Table:**

C_12_H_16_N_2_O_2_S	*F*(000) = 1072
*M**_r_* = 252.33	*D*_x_ = 1.32 Mg m^−^^3^
Orthorhombic, *P**b**c**a*	Mo *K*α radiation, λ = 0.71073 Å
Hall symbol: -P 2ac 2ab	Cell parameters from 9926 reflections
*a* = 7.2667 (5) Å	θ = 2.9–25.1°
*b* = 10.2058 (7) Å	µ = 0.25 mm^−^^1^
*c* = 34.239 (3) Å	*T* = 100 K
*V* = 2539.3 (3) Å^3^	Prism, colourless
*Z* = 8	0.37 × 0.34 × 0.23 mm

### Data collection

**Table:**

Bruker APEXII CCD diffractometer	2232 independent reflections
Radiation source: fine-focus sealed tube	2175 reflections with *I* > 2σ(*I*)
graphite	*R*_int_ = 0.019
Detector resolution: 8.333 pixels mm^-1^	θ_max_ = 25.0°, θ_min_ = 2.4°
φ and ω scans	*h* = −8→8
Absorption correction: multi-scan (*SADABS*; Sheldrick, 2008)	*k* = −12→12
*T*_min_ = 0.914, *T*_max_ = 0.946	*l* = −40→40
30412 measured reflections	

### Refinement

**Table:**

Refinement on *F*^2^	Primary atom site location: structure-invariant direct methods
Least-squares matrix: full	Secondary atom site location: difference Fourier map
*R*[*F*^2^ > 2σ(*F*^2^)] = 0.033	Hydrogen site location: inferred from neighbouring sites
*wR*(*F*^2^) = 0.072	H atoms treated by a mixture of independent and constrained refinement
*S* = 1.20	*w* = 1/[σ^2^(*F*_o_^2^) + (0.0145*P*)^2^ + 2.5358*P*] where *P* = (*F*_o_^2^ + 2*F*_c_^2^)/3
2232 reflections	(Δ/σ)_max_ = 0.001
160 parameters	Δρ_max_ = 0.25 e Å^−^^3^
0 restraints	Δρ_min_ = −0.22 e Å^−^^3^

### Special details

**Table:**

Geometry. All esds (except the esd in the dihedral angle between two l.s. planes) are estimated using the full covariance matrix. The cell esds are taken into account individually in the estimation of esds in distances, angles and torsion angles; correlations between esds in cell parameters are only used when they are defined by crystal symmetry. An approximate (isotropic) treatment of cell esds is used for estimating esds involving l.s. planes.

### Fractional atomic coordinates and isotropic or equivalent isotropic displacement parameters (Å^2^)

**Table:**

	*x*	*y*	*z*	*U*_iso_*/*U*_eq_	
S1	0.02582 (6)	0.83305 (4)	0.380695 (12)	0.01791 (12)	
O1	0.23646 (16)	0.91176 (11)	0.51723 (3)	0.0176 (3)	
O2	0.20639 (16)	1.18582 (10)	0.45356 (3)	0.0175 (3)	
N1	0.15353 (19)	0.96757 (14)	0.44114 (4)	0.0150 (3)	
N2	0.0950 (2)	1.08928 (14)	0.38546 (4)	0.0166 (3)	
C1	0.2661 (2)	1.03689 (15)	0.50384 (5)	0.0145 (3)	
C2	0.3595 (2)	1.10760 (16)	0.53071 (5)	0.0179 (4)	
H2A	0.3978	1.1963	0.5284	0.022*	
C3	0.3890 (2)	1.02359 (17)	0.56302 (5)	0.0188 (4)	
H3A	0.4499	1.0451	0.5867	0.023*	
C4	0.3138 (2)	0.90723 (17)	0.55358 (5)	0.0194 (4)	
H4A	0.3145	0.8321	0.57	0.023*	
C5	0.2064 (2)	1.07079 (15)	0.46440 (5)	0.0142 (3)	
C6	0.0933 (2)	0.97236 (16)	0.40230 (5)	0.0149 (3)	
C7	0.0241 (2)	1.11541 (16)	0.34625 (5)	0.0158 (3)	
H7A	−0.0859	1.0584	0.3418	0.019*	
C8	0.1656 (2)	1.08560 (17)	0.31451 (5)	0.0195 (4)	
H8A	0.2762	1.1408	0.3184	0.023*	
H8B	0.2035	0.9926	0.3162	0.023*	
C9	0.0824 (3)	1.11309 (17)	0.27440 (5)	0.0213 (4)	
H9A	−0.023	1.0535	0.2699	0.026*	
H9B	0.1756	1.0956	0.254	0.026*	
C10	0.0172 (2)	1.25554 (18)	0.27119 (5)	0.0223 (4)	
H10A	0.1247	1.3149	0.2726	0.027*	
H10B	−0.0434	1.269	0.2456	0.027*	
C11	−0.1176 (2)	1.28941 (17)	0.30388 (5)	0.0218 (4)	
H11A	−0.2326	1.2389	0.3002	0.026*	
H11B	−0.1486	1.3838	0.3025	0.026*	
C12	−0.0375 (2)	1.25877 (16)	0.34414 (5)	0.0175 (4)	
H12A	−0.1316	1.2756	0.3644	0.021*	
H12B	0.0689	1.3168	0.3493	0.021*	
H1	0.167 (3)	0.894 (2)	0.4510 (5)	0.021*	
H2	0.139 (3)	1.1503 (19)	0.3985 (6)	0.021*	

### Atomic displacement parameters (Å^2^)

**Table:**

	*U*^11^	*U*^22^	*U*^33^	*U*^12^	*U*^13^	*U*^23^
S1	0.0206 (2)	0.0144 (2)	0.0188 (2)	−0.00266 (17)	−0.00123 (16)	−0.00125 (16)
O1	0.0224 (6)	0.0128 (6)	0.0175 (5)	−0.0014 (5)	−0.0014 (5)	0.0024 (5)
O2	0.0222 (6)	0.0114 (6)	0.0190 (6)	0.0007 (5)	−0.0007 (5)	0.0007 (5)
N1	0.0179 (7)	0.0112 (7)	0.0159 (7)	0.0005 (6)	−0.0013 (6)	0.0024 (6)
N2	0.0196 (7)	0.0143 (7)	0.0158 (7)	−0.0012 (6)	−0.0026 (6)	−0.0005 (6)
C1	0.0139 (8)	0.0112 (8)	0.0185 (8)	0.0022 (6)	0.0037 (7)	0.0011 (6)
C2	0.0188 (8)	0.0137 (8)	0.0213 (8)	0.0013 (7)	0.0014 (7)	−0.0031 (7)
C3	0.0172 (8)	0.0234 (9)	0.0159 (8)	0.0036 (7)	−0.0010 (7)	−0.0028 (7)
C4	0.0219 (9)	0.0221 (9)	0.0141 (8)	0.0032 (7)	−0.0005 (7)	0.0036 (7)
C5	0.0101 (8)	0.0145 (8)	0.0179 (8)	0.0013 (6)	0.0026 (6)	−0.0013 (6)
C6	0.0105 (8)	0.0167 (8)	0.0174 (8)	0.0009 (6)	0.0005 (6)	0.0001 (6)
C7	0.0157 (8)	0.0159 (8)	0.0159 (8)	−0.0002 (7)	−0.0018 (7)	−0.0002 (6)
C8	0.0195 (8)	0.0181 (8)	0.0211 (8)	0.0036 (7)	0.0016 (7)	0.0005 (7)
C9	0.0236 (9)	0.0216 (9)	0.0187 (8)	0.0011 (7)	0.0032 (7)	−0.0021 (7)
C10	0.0257 (9)	0.0244 (9)	0.0169 (8)	0.0026 (8)	−0.0001 (7)	0.0038 (7)
C11	0.0251 (9)	0.0207 (8)	0.0196 (8)	0.0057 (7)	−0.0019 (7)	0.0019 (7)
C12	0.0191 (8)	0.0170 (8)	0.0165 (8)	0.0029 (7)	−0.0002 (7)	−0.0006 (6)

### Geometric parameters (Å, °)

**Table:**

S1—C6	1.6760 (16)	C7—C8	1.527 (2)
O1—C4	1.3665 (19)	C7—C12	1.532 (2)
O1—C1	1.3739 (19)	C7—H7A	1
O2—C5	1.2313 (19)	C8—C9	1.527 (2)
N1—C5	1.376 (2)	C8—H8A	0.99
N1—C6	1.401 (2)	C8—H8B	0.99
N1—H1	0.83 (2)	C9—C10	1.533 (2)
N2—C6	1.325 (2)	C9—H9A	0.99
N2—C7	1.462 (2)	C9—H9B	0.99
N2—H2	0.83 (2)	C10—C11	1.527 (2)
C1—C2	1.352 (2)	C10—H10A	0.99
C1—C5	1.460 (2)	C10—H10B	0.99
C2—C3	1.416 (2)	C11—C12	1.529 (2)
C2—H2A	0.95	C11—H11A	0.99
C3—C4	1.346 (2)	C11—H11B	0.99
C3—H3A	0.95	C12—H12A	0.99
C4—H4A	0.95	C12—H12B	0.99
			
C4—O1—C1	105.71 (13)	C9—C8—C7	109.70 (14)
C5—N1—C6	127.62 (14)	C9—C8—H8A	109.7
C5—N1—H1	115.0 (13)	C7—C8—H8A	109.7
C6—N1—H1	117.2 (13)	C9—C8—H8B	109.7
C6—N2—C7	124.08 (14)	C7—C8—H8B	109.7
C6—N2—H2	116.5 (13)	H8A—C8—H8B	108.2
C7—N2—H2	119.4 (13)	C8—C9—C10	111.18 (14)
C2—C1—O1	110.36 (14)	C8—C9—H9A	109.4
C2—C1—C5	130.70 (15)	C10—C9—H9A	109.4
O1—C1—C5	118.84 (13)	C8—C9—H9B	109.4
C1—C2—C3	106.52 (15)	C10—C9—H9B	109.4
C1—C2—H2A	126.7	H9A—C9—H9B	108
C3—C2—H2A	126.7	C11—C10—C9	111.13 (14)
C4—C3—C2	106.57 (15)	C11—C10—H10A	109.4
C4—C3—H3A	126.7	C9—C10—H10A	109.4
C2—C3—H3A	126.7	C11—C10—H10B	109.4
C3—C4—O1	110.83 (15)	C9—C10—H10B	109.4
C3—C4—H4A	124.6	H10A—C10—H10B	108
O1—C4—H4A	124.6	C10—C11—C12	111.76 (14)
O2—C5—N1	123.74 (15)	C10—C11—H11A	109.3
O2—C5—C1	120.34 (14)	C12—C11—H11A	109.3
N1—C5—C1	115.92 (14)	C10—C11—H11B	109.3
N2—C6—N1	116.20 (14)	C12—C11—H11B	109.3
N2—C6—S1	125.06 (12)	H11A—C11—H11B	107.9
N1—C6—S1	118.74 (12)	C11—C12—C7	110.44 (13)
N2—C7—C8	112.33 (13)	C11—C12—H12A	109.6
N2—C7—C12	108.69 (13)	C7—C12—H12A	109.6
C8—C7—C12	110.71 (13)	C11—C12—H12B	109.6
N2—C7—H7A	108.3	C7—C12—H12B	109.6
C8—C7—H7A	108.3	H12A—C12—H12B	108.1
C12—C7—H7A	108.3		
			
C4—O1—C1—C2	0.37 (17)	C7—N2—C6—S1	5.0 (2)
C4—O1—C1—C5	177.02 (14)	C5—N1—C6—N2	3.2 (2)
O1—C1—C2—C3	−0.65 (18)	C5—N1—C6—S1	−176.93 (13)
C5—C1—C2—C3	−176.78 (16)	C6—N2—C7—C8	−84.29 (19)
C1—C2—C3—C4	0.68 (19)	C6—N2—C7—C12	152.86 (15)
C2—C3—C4—O1	−0.47 (19)	N2—C7—C8—C9	179.24 (14)
C1—O1—C4—C3	0.08 (18)	C12—C7—C8—C9	−59.05 (18)
C6—N1—C5—O2	0.4 (3)	C7—C8—C9—C10	57.79 (19)
C6—N1—C5—C1	−179.07 (15)	C8—C9—C10—C11	−55.4 (2)
C2—C1—C5—O2	−14.7 (3)	C9—C10—C11—C12	53.9 (2)
O1—C1—C5—O2	169.45 (14)	C10—C11—C12—C7	−55.05 (19)
C2—C1—C5—N1	164.83 (17)	N2—C7—C12—C11	−178.47 (13)
O1—C1—C5—N1	−11.0 (2)	C8—C7—C12—C11	57.71 (18)
C7—N2—C6—N1	−175.11 (14)		

### Hydrogen-bond geometry (Å, °)

**Table:**

*D*—H···*A*	*D*—H	H···*A*	*D*···*A*	*D*—H···*A*
N1—H1···O1	0.83 (2)	2.329 (19)	2.7342 (17)	110.8 (16)
N1—H1···O2^i^	0.83 (2)	2.32 (2)	3.0799 (18)	153.0 (18)
N2—H2···O2	0.83 (2)	1.983 (19)	2.6574 (18)	138.0 (18)

Symmetry codes: (i) −*x*+1/2, *y*−1/2, *z*.
